# The complete mitochondrial genome of *Pycnarmon lactiferalis* (Lepidoptera: Crambidae)

**DOI:** 10.1080/23802359.2016.1214551

**Published:** 2016-09-04

**Authors:** Shan Chen, Fen-Hong Li, Xu-E Lan, Ping You

**Affiliations:** College of Life Science, Shaanxi Normal University, Xi’an, China

**Keywords:** Crambidae, mitochondrial genome, *Pycnarmon lactiferalis*

## Abstract

The complete mitochondrial genome of *Pycnarmon lactiferalis* (Walker, 1859) has been determined. The entire sequence is 15,219 bp in length which contains 13 protein-coding genes (PCGs), 22 transfer RNA genes, two ribosomal RNA genes, and an A + T-rich region. All PCGs start with the typical ATN codon except for *COI* with CGA. TAA is used for all the PCGs, while the *COI, COII* and *ND5* possess incomplete termination codons T or TA. The secondary structure of 22 tRNAs have the typical clover-leaf pattern except for *tRNA^Ser^(AGN)* lacking the dihydrouridine (DHU) stem. The A + T-rich region, located between the *srRNA* and *tRNA^Met^*, do not contain the motif “ATAGA” that is conserved across other lepidopteran species mitogenomes. A phylogenetic relationship of Pyraloidea has been reconstructed based on 13 PCGs data using Bayesian inference (BI) method.

*Pycnarmon lactiferalis* (Walker, 1859) is a species of the family Crambidae which contains various agriculture and forest pests. At present, only 23 complete mitochondrial genome sequences are available from Crambidae in GenBank database. The complete mitochondrial genome sequence of *P. lactiferalis* had been determined in this study. The new date of the sequence could provide useful information for population genetics and evolutionary studies.

Specimens of *P. lactiferalis* were collected from Xunyangba (33.33°N; 108.33°E), Ningshan County, Shaanxi province in the Qinling Mountain region of central China. DNA was extracted from the adult *P. lactiferalis*. Voucher specimens have been deposited in the Insect Collection (Accession Number SNU-Lep-20150021-5), College of Life Sciences, Shaanxi Normal University, Xi’an, China 710062.

The complete mitochondrial genome of *P. lactiferalis* is 15,219 bp in size with 81.68% A + T content. It consists of 13 protein-coding genes (PCGs), 22 transfer RNA (tRNA) genes, 2 ribosomal RNA (rRNA) genes and an A + T-rich region. Gene order is identical with other Pyraloidea species (Chai et al. 2012; Ye et al. 2013; Cao & Du 2014). There are 14 intergenic regions (except for the A + T-rich region) ranging from 1 to 47 bp in *P. lactiferalis* mitochondrial genome. The longest intergenic spacer is located between the *tRNA^Gln^* and *ND2*, with an extremely high AT content. Another intergenic spacer, located between the *tRNA^Ser^(UCN)* and *ND1*, contained the motif “ATACTAA” that is conserved across other lepidopteran species mitochondrial genomes (Cameron & Whiting 2008; He et al. 2015).

All PCGs begin with ATN codon, except for *COI*, which starts with the CGA. *COI* and *COII* genes end with T and the *ND5* gene with TA as incomplete stop codon, and the remaining genes stop with TAA. The 22 tRNA genes are ranged in size from 62 to 71 bp. The large ribosomal subunit (*lrRNA*) and small ribosomal subunit (*srRNA*) genes are 1343 and 777 bp in size, located between *tRNA^Leu^(CUN)* and AT-rich region, separated by *tRNA^Val^* gene. The length of the AT-rich region is 339 bp with 94.69% A + T content, which is between *srRNA* and *tRNA^Met^*.

A phylogenetic analysis has been performed based on the 13 PCGs dataset using BI analysis (Figure 1). A monophyly of the families Pyralidae and Crambidae is well supported. Within Crambidae, *P. lactiferalis* and other Spilomelinae species were clustered together, and this branch clustered with Pyraustinae species. The clear evolutionary relationships of them were required more taxon samples from each subfamily.

**Figure 1.**
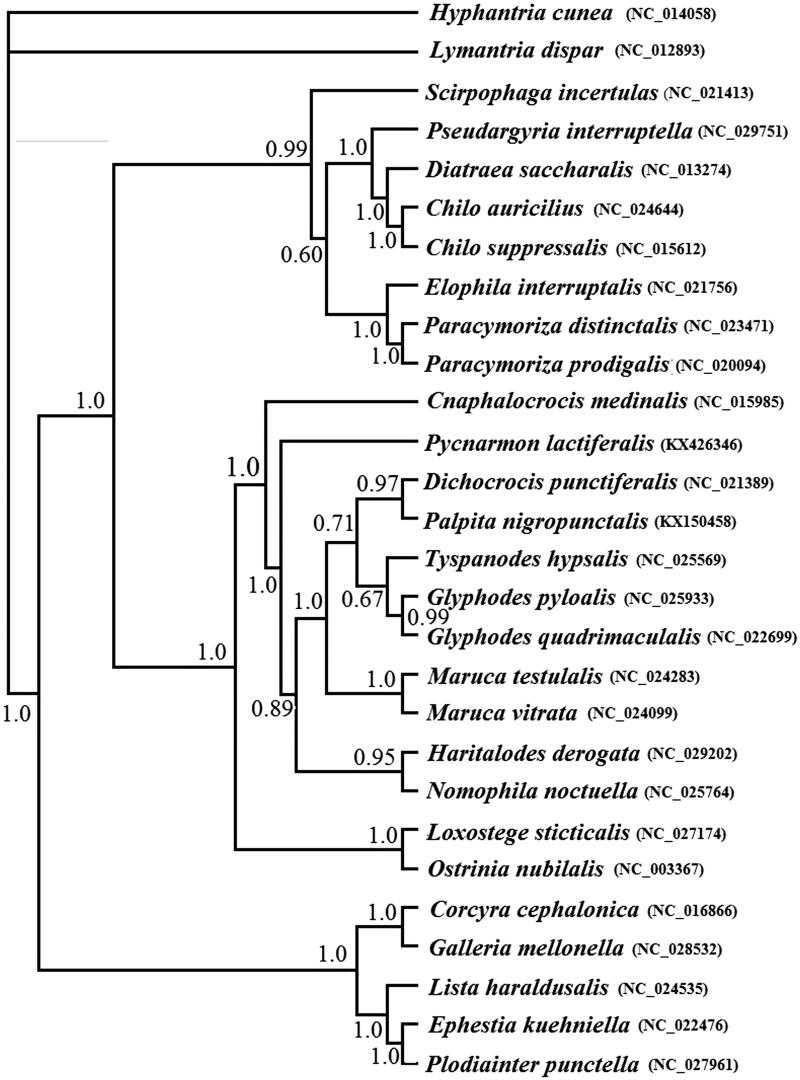
Phylogenetic tree inferred from the 13 PCGs dataset using BI analysis. (Numbers at the nodes represent Bayesian posterior probabilities respectively).

## Nucleotide sequence accession number

The complete mitochondrial genome sequence of *P. lactiferalis* has been assigned with GenBank accession number KX426346.
